# Ears for examinations: a multicentre quasi-experimental evaluation of AI-generated revision podcasts on learning outcomes and retention among medical students

**DOI:** 10.1186/s12909-026-10080-6

**Published:** 2026-08-04

**Authors:** Urvish Joshi, Amrita Sarkar, Aparajita Shukla, Sheetal Shrimali, Naba Kumar Bezbaruah, Sanjay Kini B, Sharon Baisil, Zeel Sheth, Pragya Bhargava, Stuti Shah

**Affiliations:** 1 Department of Community Medicine, Narendra Modi Medical College, Ahmedabad, Gujarat India; 2 Department of Community Medicine, Tomo Riba Institute of Health and Medical Sciences (TRIHMS), Naharlagun, Arunachal Pradesh India; 3 Department of Community Medicine, Smt NHL Municipal Medical College, Ahmedabad, Gujarat India; 4 Department of Community Medicine, Kasturba Medical College, Manipal Academy of Higher Education, Manipal, Karnataka India; 5 Department of Community Medicine, Malankara Orthodox Syrian Church Medical College, Kolenchery, Kerala India; 6 Department of Community Medicine, Swaminarayan Institute of Medical Sciences & Research, Kalol, Gujarat India

**Keywords:** Artificial intelligence, Medical education, Podcasting, Mobile learning, Mixed methods, Retention, Community medicine, India, Self-directed learning, Distributed practice

## Abstract

**Background:**

Medical curricula overload necessitates efficient, self-directed learning strategies that can utilize ‘dead time’ for study. AI-generated audio offers a scalable resource for this purpose, yet evidence regarding its efficacy in low-resource settings is limited. This study evaluated the effectiveness and learner perceptions of AI-generated podcasts on learning outcomes and retention among Indian medical students.

**Methods:**

A multicentre, quasi-experimental, mixed-methods explanatory sequential study was conducted at two geographically distinct medical colleges in India. Third-professional undergraduate MBBS students received six AI-generated, faculty-validated revision podcasts covering high-yield Community Medicine topics. Knowledge was assessed via a validated 60-item MCQ test at baseline (pre-test), immediately post-intervention, and at 30 days (delayed post-test). The intervention and assessments targeted both lower-order (remember, understand) and higher-order (apply, analyse) cognitive domains per Bloom’s Taxonomy. The primary analysis employed a Linear Mixed-Effects Model (LMM) to estimate learning trajectories. Qualitative data from Focus Group Discussions (FGDs) were analyzed using Framework Analysis to explore mechanisms of impact and integrate findings with quantitative outcomes.

**Results:**

The analytic cohort comprised 239 participants. Both centers demonstrated statistically significant immediate learning gains (Centre 1: Cohen’s d_z_ = 0.42; Centre 2: Cohen’s d_z_ = 0.55) across all levels of cognitive domain, more so in higher domain. Retention trajectories diverged: Centre 1 showed stable retention at 30 days, while Centre 2 experienced significant knowledge decay. In multivariable analysis, higher episode completion (> 50%) was independently associated with greater learning gain (adjusted β = 5.15, *p* = 0.005) after controlling for baseline scores and centre. Qualitative integration revealed that while learners valued the tool for portability, high-intensity ‘binge-listening’ at Centre 2 aligned with the observed decay, whereas distributed usage at Centre 1 supported stability. Students identified lack of pauses and AI voice monotony as key design barriers.

**Conclusions:**

The robust post-test rise validates AI-generated podcasts as an effective tool for rapid revision and pre-exam preparation. While the intervention successfully augmented study time, long-term retention favored distributed over massed practice. Future AI tools should incorporate engineered pauses and improved prosody to optimize cognitive load and sustained engagement.

**Supplementary Information:**

The online version contains supplementary material available at https://doi.org/10.1186/s12909-026-10080-6.

## Introduction

Modern medical curricula are characterized by exponential growth in knowledge and significant ‘content overload,’ which challenges students’ cognitive capacity to retain and apply information [1]. This challenge is compounded by the dynamic, ever-evolving nature of medical sciences, where guidelines and protocols update rapidly, often rendering static textbooks insufficient for keeping pace with current practice. This phenomenon is particularly acute in disciplines like Community Medicine, which requires the synthesis of complex epidemiology, clinical management, and evolving national health programs. Cognitive Load Theory posits that when the volume of information exceeds working memory capacity, learning is compromised [2, 3]. To mitigate this, educators increasingly advocate for instructional designs that facilitate spaced repetition and distributed practice, techniques known to enhance long-term retention [4]. However, the rigid schedules of traditional medical education often force ‘massed practice’ or cramming, leaving little room for structured, self-directed revision [5, 6].

In response to these constraints, mobile learning (m-learning) has emerged as a vital pedagogical tool, particularly in low-and-middle-income countries (LMICs) where smartphone penetration is high [7]. Within the m-learning ecosystem, audio podcasts have gained traction as a flexible, ‘screen-free’ modality that aligns with the principles of Free Open Access Medical Education (FOAMed). Evidence suggests that podcasts allow learners to utilize ‘dead time’-such as commuting or exercising-transforming passive downtime into active learning opportunities [8, 9]. This alignment with the ‘Modality Principle’ of multimedia learning suggests that offloading content to the auditory channel can reduce visual cognitive load, potentially enhancing engagement for fatigued students [10].

While faculty-created podcasts are effective, their production is resource-intensive, often creating a bottleneck in content generation. The recent advent of Generative AI (GenAI) offers a scalable solution, enabling the rapid synthesis of high-quality educational audio from validated texts [11]. However, this technological shift introduces a theoretical tension regarding Mayer’s ‘Voice Principle,’ which asserts that people learn better from human voices than from machine-generated ones [10]. Whether AI-generated audio content meets the pedagogical and acceptability standards expected of health professions education resources remains an evolving area of inquiry, with open questions around learner engagement, voice authenticity, and ethical use [12, 13].

Despite the proliferation of podcasts, the current literature has significant gaps. Most studies are single centre, derived from Western contexts, or focus on immediate satisfaction rather than objective retention outcomes [14, 15]. There is a paucity of multicentre data evaluating the efficacy of AI-generated audio specifically for revision in the Indian context, where the National Medical Commission has explicitly mandated the integration of digital resources to foster self-directed learning (SDL) [5, 6]. To address this, the ‘Ears for Examination’ study employed a multicentre, mixed-methods design to evaluate the effectiveness, retention durability, and learner perceptions of AI-generated revision podcasts across two geographically distinct medical colleges in India.

The specific objectives of this study were three-fold: first, to quantify the effect of the podcast intervention on immediate knowledge gain and intermediate-term retention (30 days) among undergraduate medical students, differentiating between simple recall and higher-order application; second, to identify the determinants of learning gain, specifically assessing the dose-response relationship between podcast consumption habits and assessment scores; and third, to explore learner perceptions regarding the usability, engagement, and design barriers of AI-generated audio through a qualitative framework. By integrating these quantitative outcomes with qualitative insights, the study aimed to generate evidence-based recommendations for implementing scalable, AI-assisted revision tools in resource-constrained medical education settings.

## Methods

### Study design

This study employed a multicentre, quasi-experimental study with a one-group pre-, post-, and delayed post-test design. A mixed-methods explanatory sequential approach was used, where quantitative assessment of learning outcomes and retention was followed by qualitative Focus Group Discussions (FGDs) to explore mechanisms of impact. The reporting of this study follows the TREND (Transparent Reporting of Evaluations with Nonrandomized Designs) statement for the quantitative component and GRAMMS (Good Reporting of A Mixed Methods Study) for the integration of findings (see Additional Files 1 and 2) [16, 17].

### Setting and participants

The study was conducted between August 2025 and January 2026 at two geographically and culturally distinct medical institutions in India, selected to represent diverse implementation contexts:

1. Centre 1 (West Zone of India): A medical college managed by an institutional trust under the Ahmedabad Municipal Corporation, offering undergraduate (MBBS) and postgraduate (MD) programmes. The college is affiliated with a large public teaching hospital serving a substantial and diverse catchment population from Ahmedabad city and neighbouring districts and states. The Community Medicine department delivers a competency-based curriculum (NMC framework) through traditional lectures supplemented by structured field postings at urban health training centres (UHTCs) and rural health training centres (RHTCs).
2. Centre 2 (North-East Zone of India): A fully government-funded tertiary care teaching hospital and medical sciences institute - the largest in the state - offering undergraduate (MBBS) and postgraduate (MD and DNB) programmes. It serves a predominantly tribal and semi-urban catchment population drawn from Arunachal Pradesh and neighbouring states. The Community Medicine department delivers a competency-based curriculum (NMC framework) through traditional lectures supplemented by structured field postings at urban health training centres (UHTCs) and rural health training centres (RHTCs).

The study population comprised 3rd Professional MBBS students currently rotating in Community Medicine. Recruitment and intervention occurred between August 1, 2025, and January 15, 2026. A universal sampling strategy was applied to the entire batch at both centers to minimize selection bias. Students were included if they provided digital informed consent and participated in the baseline assessment. Exclusion criteria were limited to students who declined consent or were absent during the baseline window.

To enable longitudinal tracking while maintaining strict anonymity, a self-generated identification code (SGIC) method was employed. Participants created a unique alphanumeric code based on non-sensitive personal markers (e.g., the first two letters of their mother’s name + the last two digits of their birth year). This allowed for data linkage across timepoints without collecting direct identifiers. Data cleaning involved exact matching of these codes; records with unmatchable or inconsistent identifiers were excluded from the longitudinal analysis but retained for cross-sectional components to minimize data loss.

### Intervention (The audio podcast)

The intervention, titled ‘Ears for Examinations’, consisted of a series of six AI-generated, faculty-validated revision podcasts. The development and delivery of the intervention are described below in accordance with the TIDieR (Template for Intervention Description and Replication) checklist (see Additional File 3) [18]: [18]

- Format and Content: Each episode was structured as a conversational dialogue between an anchor and a senior professor of Community Medicine, moving from basic recall (definitions/epidemiology) to higher-order application (case scenarios and management). The series comprised 6 episodes on 6 different subjects, covering high-yield Community Medicine topics: Iron Deficiency Anemia, Malaria, HIV/AIDS, Hypertension, Cardiovascular Diseases, and Integrated Child Development Services. These topics were purposefully selected as they had already been covered in didactic theory lectures, positioning the intervention explicitly as a reinforcement and revision tool rather than a primary teaching method.
- Duration: Each episode was designed as a ‘high-yield summary’ with a duration of 8–10 min, optimized for short attention spans.
- Production: Core content was synthesized from standard textbooks and national guidelines. The scripts were prepared and finalized manually, utilizing Large Language Models (LLMs) for formatting assistance, and underwent mandatory validation by two senior Community Medicine faculty members to ensure medical accuracy and alignment with the National Medical Commission (NMC) curriculum. Validation involved a structured two-round review: faculty independently assessed each script against a checklist covering factual accuracy, curriculum alignment, and cognitive level appropriateness, followed by a consensus meeting to resolve disagreements before scripts were locked for audio production. Once scripts were locked, the AI generation process-including text-to-speech conversion using high-fidelity engines (e.g., Google AI Studio, ElevenLabs) and YouTube hosting with visual assets-took approximately 30–45 min per episode, demonstrating significant time efficiency compared to traditional recording methods.
- Access and Delivery: The episodes were hosted on a private YouTube channel to ensure universal accessibility across devices. Students were provided with links via their batch WhatsApp groups. All six episodes were made available simultaneously at the start of the 15-day intervention window, allowing students to self-direct the order and pace of consumption.
- Mode of Delivery: The intervention was asynchronous and self-directed. The intervention window lasted 15 days, during which students were encouraged to listen to the episodes at their own pace, including during ‘dead time’ (e.g., commuting, breaks).
- Adherence was monitored using aggregate platform analytics (cumulative views and average watch time on YouTube) and triangulation with self-reported frequency of listening in the post-intervention survey.

### Data collection

Data collection employed a mixed-methods approach. Quantitative data were collected via online forms (Google Forms) at three distinct timepoints:

1. Pre-test (Baseline): Administered immediately prior to the release of the podcasts to assess baseline knowledge and attitudes before any exposure.
2. Post-test (Immediate): Administered immediately after the 15-day intervention window to assess immediate knowledge gain. This form also included a Usage and Perception Survey collecting self-reported ‘dose’ (episodes and duration completed), listening context (e.g., commute vs. study desk), feedback on the AI-generated content, and concurrent consumption of other educational resources on the same topics to adjust for potential confounding. The survey items were face-validated by faculty and pilot-tested with a small group of interns (*n* = 10) for clarity prior to deployment.
3. Delayed Post-test (Retention): Administered approximately 4 weeks (30 days) after the post-test to assess (intermediate term) retention. This interval was selected to align with the typical duration of clinical rotations and the subsequent internal assessment cycle.

Qualitative data from Focus Group Discussions were captured using a dual-device audio recording protocol to ensure redundancy, supplemented by field notes on non-verbal cues taken by a designated scribe.

### Outcome measures

#### Quantitative learning outcomes

The primary instrument was a 60-item Multiple Choice Question (MCQ) test, blueprinted to ensure content validity, and designed to facilitate discrimination between high and low performers by including a balanced mix of difficulty levels (Easy: 30%, Moderate: 40%, Difficult: 30%) per topic, though formal item response theory (IRT) analysis was not performed. Item difficulty levels were assigned a priori by consensus of two senior faculty members during blueprinting, guided by each item’s cognitive demand and alignment with NMC curriculum objectives. All 60 items were newly developed for this study and had not been previously used in formal assessments. The test structure included 10 items per topic for each of the six intervention topics.

- Cognitive Domains: Items were mapped to Bloom’s taxonomy: Recall (40%; 4 items/topic), Application (30%; 3 items/topic), and Analysis (30%; 3 items/topic). This distribution, ensuring that 60% of the test assessed higher-order thinking, was intentionally selected to measure the intervention’s ‘value-add’ in fostering clinical reasoning and application beyond simple rote memorization.
- Content Alignment: Test items were blueprinted to align with the specific learning objectives covered in the intervention scripts. To minimize cueing and ensure valid assessment of understanding rather than mere recall, case scenarios in the assessment differed substantially from those used in the podcasts (e.g., changing patient demographics, clinical values, or context).
- Scoring: Each correct response was awarded 1 mark (possible score range: 0–60).
- Reliability: The same 60-item bank was used across all timepoints with item shuffling to maintain measurement equivalence. Internal consistency reliability (Cronbach’s alpha) was calculated at each timepoint.

#### Qualitative data

Focus Group Discussions (FGDs) were conducted at both centres following the post-test phase (3 FGDs at Centre 1 and 1 FGD at Centre 2). The sessions utilized a semi-structured interview guide developed specifically for this study (See Additional File 4) to explore learning mechanisms, usability barriers (e.g., AI voice quality), and usage behaviours. The number of FGDs was proportional to the batch size and determined by the point of thematic saturation; the single in-depth session at Centre 2 was sufficient to confirm thematic concordance with the primary site. The sessions were led by a faculty moderator with a dedicated scribe noting non-verbal cues. To prevent data loss, a redundant audio recording protocol (primary and backup devices on Airplane Mode) was used. Immediately post-session, a ‘Hot Note’ debrief was conducted to capture initial impressions (See Additional File 4).

FGDs were conducted in English, the medium of clinical instruction at both sites; where participants occasionally expressed themselves in regional or vernacular terms, these were translated into English during the mandatory manual transcript verification process by a bilingual team member at the respective centre. At Centre 1, sessions were led by the first author (UJ) as lead moderator with ZS as co-moderator; PB and SS served as designated scribes. At Centre 2, sessions were moderated by AS, with a designated member of the research team serving as scribe. Participants for the qualitative arm were purposively sampled from students who had completed the post-test, with deliberate inclusion of a mix of high, moderate, and low episode-completion engagers to ensure a range of perspectives. In addition to written digital consent obtained for the quantitative component, separate verbal and written informed consent for audio recording was obtained from each FGD participant immediately prior to session commencement. 

##### Integration

A contiguous approach was used where qualitative data were analyzed after quantitative results to explain observed patterns (explanatory sequential). Integration occurred at the interpretation stage using a joint display matrix, where quantitative retention trajectories (e.g., ‘Decay at Centre 2’) were juxtaposed with qualitative themes (e.g., ‘Binge-listening behaviour’) to generate meta-inferences.

### Statistical analysis

Quantitative data were analyzed using SPSS v27 (IBM Corp., Armonk, NY, USA) [19].

### Power analysis

Although a universal sampling strategy was employed - precluding a priori sample-size determination as a recruitment criterion - a retrospective adequacy check confirmed that the final analytic sample (*N* = 239) substantially exceeded the threshold of 120 participants needed to detect a medium effect size (Cohen’s d = 0.5) with 80% power (alpha = 0.05), providing adequate statistical precision for multicentre outcome estimation.

### Primary analysis

To account for the hierarchical nature of the data (repeated measures nested within participants) and the multicentre design, a Linear Mixed-Effects Model (LMM) was used as the primary analysis.

- Dependent Variable: Total MCQ Score.
- Fixed Effects: Timepoint (Pre/Post/Delayed), Centre (1 vs. 2), and their interaction (Timepoint × Centre). ‘Centre’ was treated as a fixed effect rather than a random effect due to the small number of macro-units (*n* = 2), allowing for direct estimation of institutional differences.
- Random Effect: Participant intercept (StudyID) to account for within-person correlation.
- Estimated marginal means (EMMeans) were calculated, and planned contrasts (Holm-adjusted) were used to assess immediate gain (Pre vs. Post) and retention/decay (Post vs. Delayed).

#### Engagement metrics

A multiple linear regression model was constructed to analyze the association between ‘dose’ and ‘learning gain’ (Post − Pre score), controlling for baseline score and centre. To mitigate recall bias inherent in minute-level self-reporting, ‘dose’ was operationalized as a binary variable (> 50% episode completion vs. <50%).

#### Secondary analyses

- Paired Comparisons: For the subset of ‘completers’ (participants with linked data at specific timepoints), paired t-tests were used to calculate effect sizes (Cohen’s d_z_). Attrition analysis was performed to compare baseline characteristics (scores, gender) between retained participants and dropouts to assess potential selection bias.
- Robustness Checks: Non-parametric Wilcoxon signed-rank tests were conducted to verify that statistical significance was not driven by distributional violations.

### Qualitative analysis and integration

Initial transcripts were drafted using AI-assisted tools (e.g., Otter.ai) to ensure efficiency, followed by a mandatory manual verification process where investigators listened to the original audio to correct errors and ensure the accuracy of medical terminology. The verified transcripts were analyzed using Framework Analysis, chosen for its structured approach to policy-relevant questions and ability to facilitate transparent cross-case comparison in multicentre designs [20]. Data were coded and mapped onto a matrix to identify key themes regarding utility, engagement, and design barriers. To ensure analytical rigour, a reflexive stance was maintained throughout the coding process: while moderators explicitly encouraged critical feedback, the potential for social desirability bias stemming from their faculty positions was acknowledged and accounted for during interpretation. Mixed-methods integration was achieved using a Joint Display Analysis, where quantitative retention trends were juxtaposed with qualitative explanatory themes to generate meta-inferences.

## Results

### Participant flow and dataset completeness

**Fig. 1:**
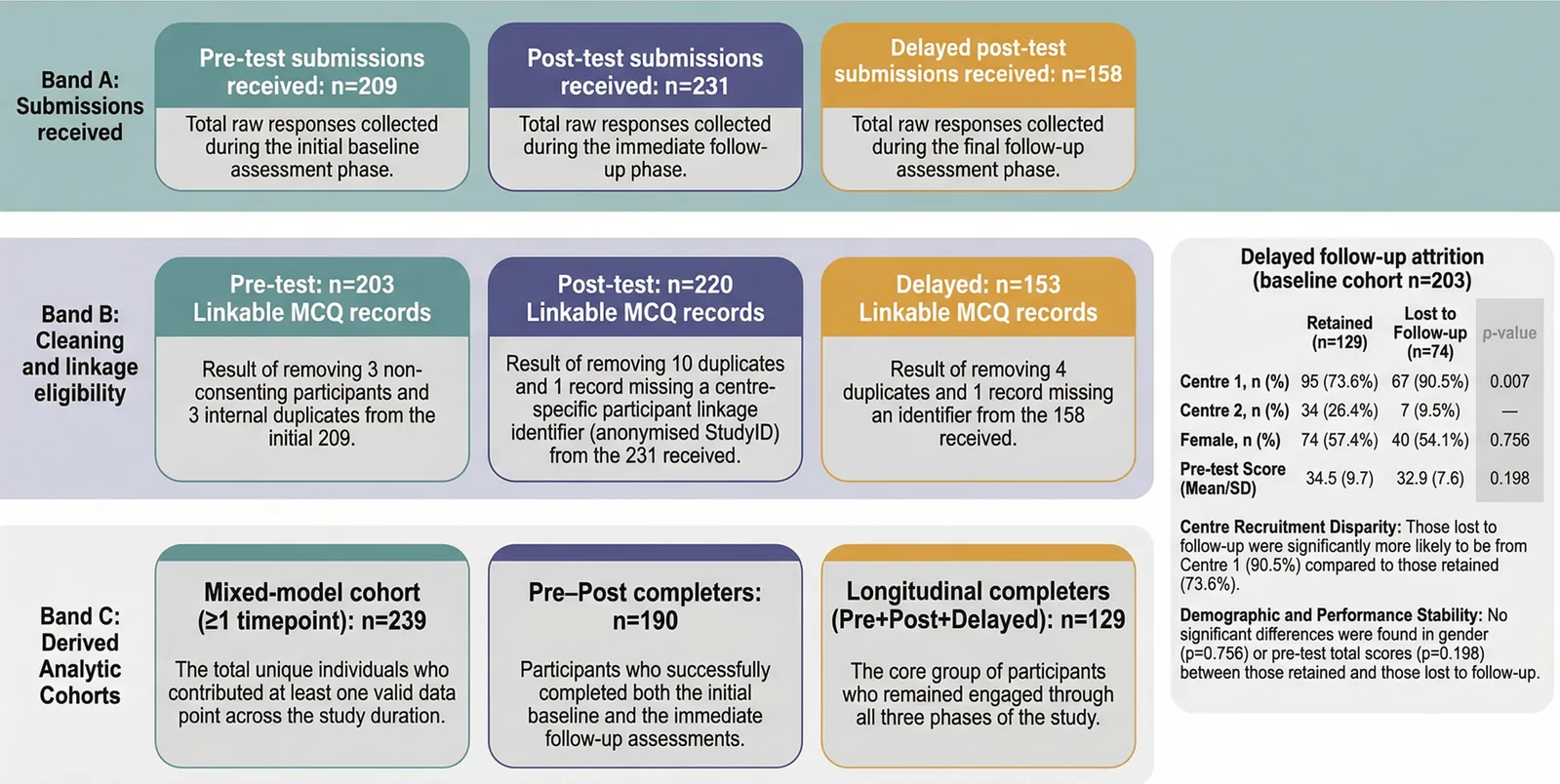
Participant flow diagram

Participant flow and derivation of analytic cohorts are shown in Fig. 1. We received 209 pre-test, 231 post-test, and 158 delayed post-test submissions. After consent screening, within-timepoint deduplication, and excluding records without a centre-specific participant linkage identifier for longitudinal linkage, the linkable MCQ datasets comprised 203 pre-test, 220 post-test, and 153 delayed records. From these, 239 unique participants contributed at least one timepoint (mixed-model cohort), 190 were pre–post completers, and 129 completed all three timepoints. Delayed follow-up attrition from the baseline cohort (*n* = 203) was 74/203 and was primarily driven by centre (Centre 1: 90.5% of those lost vs. 73.6% of those retained), while gender and baseline score showed minimal differences. Reliability and post-test survey analyses used all complete submissions after consent screening and within-timepoint deduplication (post-test *n* = 221; delayed *n* = 154), regardless of linkage eligibility.

### Baseline characteristics

Baseline characteristics are summarised in Table 1 (*N* = 203; Centre 1 *n* = 162, Centre 2 *n* = 41). Gender distribution was similar across centers. Baseline MCQ performance differed by centre, with Centre 2 showing higher pre-test total scores (38.8 (13.8) vs. 32.7 (6.9); *p* = 0.009) and higher recall sub score (15.7 (5.3) vs. 13.2 (3.1); *p* = 0.005). Follow-up completion also differed, with a higher proportion of Centre 2 participants completing all three timepoints (82.9% vs. 58.6%; *p* = 0.007).

**Table 1:** Baseline ceristiacs by centrharcte

Characteristic	Overall	Centre 1	Centre 2	*p*-value*
Male, *n* (%)	89 (43.8)	71 (43.8)	18 (43.9)	1
Female, *n* (%)	114 (56.2)	91 (56.2)	23 (56.1)	
Pre-test total score - Mean (SD)	33.9 (8.7)	32.7 (6.9)	38.8 (13.8)	0.009
Recall sub score (0–24) - Mean (SD)	13.7 (3.8)	13.2 (3.1)	15.7 (5.3)	0.005
Higher-order sub score (0–36) - Mean (SD)	20.2 (5.9)	19.5 (4.8)	23.0 (9.0)	0.018
Completed Pre+Post, *n* (%)	190 (93.6)	154 (95.1)	36 (87.8)	0.181
Completed longitudinal, *n* (%)	129 (63.5)	95 (58.6)	34 (82.9)	0.007

Legend: *N* = 203. * Comparisons by chi-square (gender, completion rates) and Welch t-test (score variables). Recall: Bloom’s recall-domain items; Higher-order: Application + Analysis items combined

Internal consistency of the MCQ instrument was high and improved across timepoints: Cronbach’s alpha was 0.85 at pre-test (*N* = 203), 0.91 at post-test (*N* = 221), and 0.94 at the delayed post-test (*N* = 154), consistent with increasing score variance following learning rather than any change in item quality.

### Primary outcome: learning gain and retention (LMM)

Model-estimated trajectories and Holm-adjusted planned contrasts are presented in Table 2. Centre 1 increased from 32.3 (pre-test) to 35.8 (post-test) and remained above baseline at delayed follow-up (34.6), while Centre 2 increased from 38.2 to 45.0 with partial decline at delayed (41.0). Immediate pre–post gains were statistically clear in both centers (Holm-adjusted *p* < 0.001), while post–delayed change was significant only at Centre 2 (Holm-adjusted *p* = 0.033).

**Table 2:** Model-estimated score trajectories and learning gains (LMM)

Centre	Timepoint	Estimated mean score (95% CI)	Comparison	Mean difference (95% CI)	Adj. *p*-value
Centre 1	Pre-test	32.3 (30.6 to 33.9)	Reference	-	-
	Post-test	35.8 (34.3 to 37.4)	Pre vs. Post (Immediate gain)	3.6 (1.9 to 5.2)	*p* < 0.001
	Delayed	34.6 (32.7 to 36.5)	Post vs. Delayed (Decay)	-1.2 (-3.2 to 0.7)	0.206
			Pre vs. Delayed (Net retention)	2.3 (0.4 to 4.3)	0.037
Centre 2	Pre-test	38.2 (35.0 to 41.4)	Reference	-	-
	Post-test	45.0 (41.9 to 48.1)	Pre vs. Post (Immediate gain)	6.8 (3.4 to 10.1)	*p* < 0.001
	Delayed	41.0 (37.9 to 44.1)	Post vs. Delayed (Decay)	-4.0 (-7.3 to -0.7)	0.033
			Pre vs. Delayed (Net retention)	2.8 (-0.6 to 6.1)	0.108

Legend: Linear mixed-effects model (TotalScore ~ Timepoint + Centre + Timepoint×Centre + (1|StudyID); REML). Estimated marginal means (95% CI) shown. Contrasts are within-centre and Holm-adjusted. Full fixed-effect estimates in Additional File 5, Table S1

Full fixed-effect estimates from the primary mixed-effects model are provided in Table S1 (See Additional File 5).

### Secondary outcomes (paired replication; domain; topic-exploratory)

Paired within-participant analyses among linked completers are presented in Table 3. Immediate learning gains from pre-test to post-test were observed at both centers, with mean differences of 3.9 at Centre 1 (d_z_ 0.42, *p* < 0.001) and 7.3 at Centre 2 (d_z_ 0.55, *p* = 0.002). Post-test to delayed follow-up changes were smaller and less conclusive in paired completers (Centre 1 mean difference − 1.3, *p* = 0.109; Centre 2 -3.5, *p* = 0.102). Net gain from pre-test to delayed follow-up remained positive, particularly at Centre 1 (mean difference 2.7, *p* = 0.025).

**Table 3:** Paired analysis by completer cohort (legacy replication)

Outcome variable	Centre	Timepoint 1 Mean (SD)	Timepoint 2 Mean (SD)	Mean difference (95% CI)	Effect size (d_z_)	*p*-value
Immediate gain (Pre vs. Post)	Centre 1 (*n* = 154)	32.7 (6.9)	36.6 (10.6)	3.9 (2.4 to 5.4)	0.42	*p* < 0.001
	Centre 2 (*n* = 36)	39.1 (13.5)	46.4 (9.7)	7.3 (2.8 to 11.8)	0.55	0.002
Retention/decay (Post vs. Delayed)	Centre 1 (*n* = 95)	36.8 (10.5)	35.4 (12.9)	-1.3 (-3.0 to 0.3)	-0.17	0.109
	Centre 2 (*n* = 34)	46.1 (9.7)	42.6 (14.6)	-3.5 (-7.8 to 0.7)	-0.29	0.102
Net gain (Pre vs. Delayed)	Centre 1 (*n* = 95)	32.7 (7.1)	35.4 (12.9)	2.7 (0.3 to 5.1)	0.22	0.025
	Centre 2 (*n* = 34)	39.4 (13.7)	42.6 (14.6)	3.2 (-1.4 to 7.8)	0.24	0.166

Legend: Paired t-tests in linked completers. Pre–Post completers: C1 *n* = 154, C2 *n* = 36. Longitudinal completers (all three timepoints): C1 *n* = 95, C2 *n* = 34. Cohen’s dz is the paired effect size

Domain-specific changes are shown in Table 4. Both centers improved in Recall and Higher-order domains. For Recall, Centre 1 improved by 1.5 points (Holm-adjusted *p* < 0.001), while Centre 2 improved by 3.3 points (Holm-adjusted *p* < 0.001). Higher-order gains were also evident (Centre 1 2.4, Holm-adjusted *p* < 0.001; Centre 2 4.0, Holm-adjusted *p* = 0.013), indicating improvement beyond simple recall.

**Table 4:** Domain-specific analysis (Bloom’s taxonomy; exploratory)

Domain	Centre	Pre mean (SD)	Post mean (SD)	Mean difference (95% CI)	Effect size (d_z_)	Adj. *p*-value
Recall (Lower order)	Centre 1 (*n* = 154)	13.3 (3.1)	14.7 (4.5)	1.5 (0.8 to 2.1)	0.35	*p* < 0.001
	Centre 2 (*n* = 36)	15.7 (5.1)	19.0 (3.9)	3.3 (1.6 to 5.0)	0.66	*p* < 0.001
Higher order (Application+Analysis)	Centre 1 (*n* = 154)	19.4 (4.9)	21.9 (6.6)	2.4 (1.4 to 3.5)	0.39	*p* < 0.001
	Centre 2 (*n* = 36)	23.3 (8.9)	27.3 (6.4)	4.0 (0.9 to 7.1)	0.43	0.013

Legend: Paired t-tests within pre–post completers. Holm adjustment across two domains per centre. Domain mapping follows the MCQ answer key (see Figshare data repository)

Exploratory topic-wise performance is reported in Table 5. Gains were heterogeneous across topics and centers. At Centre 1, the largest improvements were in HIV (absolute gain 12.9%, Holm-adjusted *p* < 0.001) and HTN (8.8%, Holm-adjusted *p* < 0.001), with smaller improvements in several other topics. At Centre 2, improvements were seen across most topics (e.g., CVD 16.1%, Holm-adjusted *p* = 0.022; HIV 15.8%, Holm-adjusted *p* = 0.003), while IDA showed no meaningful change (0.3%, Holm-adjusted *p* = 0.947).

**Table 5:** Topic-wise performance

Topic	Centre	Pre % correct	Post % correct	Absolute gain (%, 95% CI)	Effect size (d_z_)	Adj. *p*-value
IDA	Centre 1	47	51.3	4.3 (0.6 to 8.0)	0.19	0.056
	Centre 2	70	70.3	0.3 (-8.1 to 8.6)	0.01	0.947
ICDS	Centre 1	49.6	53.6	4.0 (0.6 to 7.4)	0.19	0.056
	Centre 2	59.7	71.4	11.7 (1.9 to 21.4)	0.42	0.023
CVD	Centre 1	54	58.2	4.2 (0.6 to 7.7)	0.19	0.056
	Centre 2	59.4	75.6	16.1 (5.4 to 26.9)	0.54	0.022
Malaria	Centre 1	67.9	72.8	4.9 (1.5 to 8.3)	0.22	0.024
	Centre 2	72.8	86.1	13.3 (3.3 to 23.4)	0.45	0.023
HTN	Centre 1	56.1	64.8	8.8 (4.7 to 12.8)	0.35	*p* < 0.001
	Centre 2	60.3	76.1	15.8 (5.0 to 26.7)	0.47	0.022
HIV	Centre 1	52.3	65.2	12.9 (9.0 to 16.8)	0.51	*p* < 0.001
	Centre 2	68.3	84.2	15.8 (7.1 to 24.6)	0.62	0.003

Legend: Topic scores expressed as percent correct across 10 items per topic. Paired t-tests within pre–post completers; Holm adjustment across six topics per centre. Topic-wise results are exploratory

### Learner perceptions and usage

Post-test survey responses are summarised in Table 6 (*N* = 221; Centre 1 *n* = 176, Centre 2 *n* = 45). Engagement differed by centre: a higher proportion of learners at Centre 2 reported listening to 5–6 episodes (35.6%) compared with Centre 1 (15.3%) (overall distribution *p* < 0.001). Listening completeness also favoured Centre 2, with more learners reporting listening to the full episode (64.4% vs. 35.2%, *p* = 0.006). Listening context varied (*p* < 0.001), while device use was similar across centers and predominantly mobile-based. Overall perceptions were favourable: mean ratings for perceived recall support and AI voice clarity were around the positive mid-to-high range, and recommendation intent (NPS 0–10) was comparable across centres.

**Table 6:** Usage patterns and student satisfaction survey (descriptive)

Survey item	Overall (*N* = 221)	Centre 1 (*N* = 176)	Centre 2 (*N* = 45)	*p*-value
Episodes listened (of 6)				*p* < 0.001
0	26 (11.8)	25 (14.2)	1 (2.2)	
1–2	79 (35.7)	71 (40.3)	8 (17.8)	
3–4	73 (33.0)	53 (30.1)	20 (44.4)	
5–6	43 (19.5)	27 (15.3)	16 (35.6)	
Average listening per episode				0.006
Didn’t listen to any	27 (12.2)	26 (14.8)	1 (2.2)	
Less than half	41 (18.6)	36 (20.5)	5 (11.1)	
More than half	56 (25.3)	47 (26.7)	9 (20.0)	
The full episode	91 (41.2)	62 (35.2)	29 (64.4)	
Full episode multiple times	6 (2.7)	5 (2.8)	1 (2.2)	
Primary listening context				*p* < 0.001
During free time in college	71 (32.1)	57 (32.4)	14 (31.1)	
Dedicated study time at home	34 (15.4)	20 (11.4)	14 (31.1)	
While commuting/traveling	37 (16.7)	35 (19.9)	2 (4.4)	
While doing chores/daily activities	34 (15.4)	31 (17.6)	3 (6.7)	
Just before sleep	24 (10.9)	13 (7.4)	11 (24.4)	
Did not listen	21 (9.5)	20 (11.4)	1 (2.2)	
Device used most often				1.000†
Smartphone + headphones/earbuds	149 (67.4)	122 (69.3)	27 (60.0)	
Smartphone speaker	36 (16.3)	26 (14.8)	10 (22.2)	
Tablet	29 (13.1)	22 (12.5)	7 (15.6)	
Laptop/Desktop	7 (3.2)	6 (3.4)	1 (2.2)	
Used online coaching apps/YouTube (Yes)	88 (39.8)	74 (42.0)	14 (31.1)	0.243
‘Podcasts helped recall better’ (1–5)	3.4 (1.1)	3.4 (1.1)	3.4 (1.1)	0.942‡
‘Revising felt less boring’ (1–5)	3.6 (1.1)	3.6 (1.1)	3.4 (1.2)	0.388‡
‘AI voice was clear’ (1–5)	3.6 (1.1)	3.6 (1.1)	3.6 (1.2)	0.962‡
Recommend to juniors (NPS 0–10)	6.7 (2.5)	6.7 (2.4)	6.7 (2.8)	0.893‡

Legend: Post-test survey (*N*=221 after within-timepoint deduplication). Values: *n* (%) or mean (SD). Chi-square for multi-category distributions; † Fisher’s exact test for device use (sparse cells); ‡ Mann-Whitney U test for Likert and NPS items

### Predictors of learning gain

Predictors of learning gain among linked pre–post completers are shown in Table 7. Baseline score was inversely associated with gain (β -0.50, *p* < 0.001), consistent with less headroom for improvement among higher baseline performers. In univariable analysis, Centre 2 was associated with higher learning gains. However, in the final multivariable model adjusting for episode completion (‘dose’), the direct effect of Centre on learning gain was attenuated and no longer statistically significant (adjusted β = 5.22, *p* = 0.485; see Table 7), suggesting that the performance difference was mediated by differential engagement. Using a conservative definition of > 50% episode completion (listening to 5–6 episodes), higher episode completion was independently associated with greater learning gain (β 5.15, *p* = 0.005). Overall model fit was moderate (R^2^ 0.233, adjusted R^2^ 0.195).

**Table 7:** Predictors of learning gain (multivariable regression)

Predictor	Unstandardized β (SE)	Standardized β	95% CI for β	*p*-value
(Constant)	12.63 (5.26)	-	2.26 to 23.01	0.017
Baseline				
Pre-test total score	-0.50 (0.08)	-0.43	-0.65 to -0.34	*p* < 0.001
Centre 2 (Ref: Centre 1)	5.22 (1.82)	0.2	1.62 to 8.82	0.005
Usage / context				
Episodes completed (> 50%)*	5.15 (1.79)	0.21	1.61 to 8.68	0.005
Listening context (Commute vs. Home)	-1.30 (1.81)	-0.05	-4.88 to 2.28	0.475
Device used (Mobile vs. Laptop)	4.07 (3.86)	0.07	-3.56 to 11.69	0.294
Used other coaching apps (Yes/No)	0.16 (1.43)	0.01	-2.67 to 2.98	0.912
Feedback ratings (Likert 1–5)				
Perceived usefulness	0.15 (1.10)	0.02	-2.01 to 2.31	0.89
Audio quality rating	1.12 (0.89)	0.12	-0.64 to 2.88	0.213
Content engagement rating	-0.44 (1.04)	-0.05	-2.49 to 1.61	0.674

Legend: Outcome = learning gain (Post − Pre total score) among linked pre–post completers (*N* = 190). * >50% episode completion = 5–6 episodes listened. Model fit: R²=0.233, adjusted R²=0.195

### Qualitative findings

Three FGDs were conducted at Centre 1 (*n* = 8, *n* = 7, and *n* = 6 participants respectively; total *n* = 21) and one in-depth FGD at Centre 2 (*n* = 9 participants), giving a combined qualitative sample of 30 students. Participants varied across gender and episode-completion levels, consistent with the purposive sampling approach. Session duration ranged from 45 to 70 min at Centre 1 and was 60 min at Centre 2. Thematic saturation was assessed iteratively; the single session at Centre 2 confirmed concordance with themes from Centre 1 without yielding substantially new themes.

Framework analysis identified three overarching themes, consistent across both centres. Theme 1 - Revision utility and portability: Participants framed the podcasts as effective tools for reinforcing content already covered in lectures, particularly valued for enabling revision during ‘dead time’ such as commuting or college free periods. Subthemes included fit with pre-examination revision workflows and hands-free multitasking utility. Representative quote: ‘…could mentally revise the whole chapter’ (Centre 2, P8). Theme 2 - Engagement and design barriers: While overall AI voice clarity was acceptable (mean rating 3.6/5), the continuous, uninterrupted audio format was identified as a source of cognitive fatigue. Participants requested deliberate pauses after case scenarios and between question-answer segments to facilitate active retrieval. AI voice monotony was noted as a barrier to sustained attention. Subthemes included AI voice naturalness and absence of retrieval-practice prompts. Representative quotes: ‘there was no pause in between’ (Centre 1, FGD1, P1); ‘quite robotic!’ (Centre 2, P4). Theme 3 - Reinforcement behaviours and multimodal needs: Participants reporting stable retention described active reinforcement habits such as note-taking while listening and voluntary re-listening. Learners at both centres requested multimodal companions - brief key-point PDFs, infographics, or embedded self-check questions - to anchor audio-based learning. Subthemes included notetaking as an active retrieval strategy and desire for visual anchors. Representative quote: ‘because I like to make notes when I listen’ (Centre 1, FGD3, P3). A detailed theme-subtheme framework with centre-wise patterns and additional exemplar quotes is provided in Supplementary Table S3 (Additional File 5). These themes are integrated with quantitative findings in the joint display.

In summary, the intervention produced consistent immediate learning gains across both institutional contexts, with the key differentiator being retention: distributed usage at Centre 1 supported durable gains at 30 days, while high-intensity usage at Centre 2 was associated with significant attenuation. Across both quantitative and qualitative strands, higher engagement with the episodes was the principal driver of outcome, and learner-identified design gaps - particularly the absence of deliberate pauses and multimodal anchors - point to actionable refinements for future iterations.

A detailed theme–subtheme framework with centre-wise patterns and exemplar quotes is provided in Supplementary Table S3 (See Additional File 5).

### Mixed-methods integration (Joint display)

Mixed-methods integration is presented in Table 8. Quantitative and qualitative findings were largely convergent: measured learning gains at both centres (Table 2) aligned with learners describing the podcasts as useful for revision and recall, while the observed post-to-delayed attenuation-more prominent at Centre 2-aligned with comments emphasizing the need for reinforcement and revisit-friendly study habits (e.g., note-taking while listening). Overall, learners valued the format for opportunistic study during travel or breaks, alongside practical recommendations to improve pacing and voice naturalness.

**Table 8:** Joint display (mixed-methods integration)

Quantitative finding (where reported)	Qualitative theme (centre)	Exemplar quote (de-identified)	Meta-inference (integration)
Immediate learning gains at both centres (Tables 2 and 3)	Revision and recall support (Centre 2)	‘trigger memory of revising and active material more recall’	Objective gains were congruent with perceived usefulness for revision and retrieval.
Higher engagement at Centre 2: 5–6 episodes listened 35.6% vs. 15.3%; full-episode listening 64.4% vs. 35.2% (Table 6)	Convenient, hands-free learning during ‘dead time’ (Centre 2)	‘hands-free… could mentally revise… breaks, travel, the whole chapter’	Higher uptake aligned with learners’ emphasis on multitasking-friendly revision opportunities.
Post-to-delayed attenuation, particularly at Centre 2 (Tables 2 and 3)	Reinforcement and revisit-friendly learning (Centre 1)	‘because I like to make notes when I listen’	Where retention attenuated, learners highlighted reinforcement behaviours (e.g., note-taking) as part of effective use.
Overall voice clarity rated positively (mean 3.6/5), but design refinements suggested (Table 6)	Pacing and voice naturalness shape attention (Centres 1 and 2)	‘there was no pause in between’ (Centre 1); ‘quite robotic!’ (Centre 2)	Small acceptability frictions may reduce sustained attention; optimizing pacing and prosody may support engagement and retention.

Legend: Joint display integrating quantitative outcomes (Tables 2 and 3), usage patterns (Table 6), and qualitative themes from FGDs at both centres. Quotes are verbatim and de-identified. Meta-inferences reflect convergence of measured learning patterns and learner-reported mechanisms

### Sensitivity/robustness

Non-parametric Wilcoxon signed-rank sensitivity checks for paired comparisons were consistent in direction with paired t-tests, with occasional differences in statistical conclusiveness (Table S2, Additional File 5). Specifically, the post-to-delayed comparison at Centre 1 and the pre-to-delayed comparison at Centre 1 showed directional agreement but differed marginally in significance thresholds between the two methods, reflecting the smaller Centre 1 completer subsample (*n* = 95) rather than any reversal of effect direction.

## Discussion

### Principal findings

This multicentre quasi-experimental evaluation found that AI-generated, faculty-validated revision podcasts were associated with significant immediate learning gains across two geographically distinct institutional contexts, confirming the intervention’s efficacy as a revision tool. Critically, gains extended beyond simple recall to higher-order cognitive domains - application and analysis - suggesting the intervention can support clinical reasoning when content is scripted effectively, though topic-specific performance was variable. The principal differentiator was retention: Centre 1 maintained stable knowledge scores at 30 days, while Centre 2 experienced significant post-to-delayed attenuation. This divergence was not explained by institutional factors alone; in multivariable analysis, the direct effect of centre on learning gain was attenuated and no longer statistically significant (*p* = 0.485) after controlling for episode completion (‘dose’), pointing to implementation behaviour - specifically how students used the podcasts - as the primary driver. Measurement reliability was high throughout, with Cronbach’s alpha reaching 0.94 at the delayed assessment, supporting confidence in the outcome data.

### Interpretation and mechanisms

These results suggest an association between AI-generated audio revision and immediate learning gains in a pragmatic setting, though the divergence in retention trajectories warrants careful interpretation. While Centre 2 participants initially outperformed Centre 1, the adjusted multivariable analysis indicates that this difference was largely mediated by engagement levels; when controlling for ‘dose’ (episode completion), the direct effect of ‘Centre’ on learning gain was no longer statistically significant (*p* = 0.485; Table 7). This finding is consistent with a dose-response relationship (β = 5.15, *p* < 0.001), suggesting that the intervention’s utility is driven by the intensity of usage rather than institutional factors alone.

The contrasting retention patterns-rapid decay at Centre 2 versus stability at Centre 1-may be interpreted through the lens of cognitive psychology. The high-intensity, clustered usage observed at Centre 2 mirrors a ‘massed practice’ phenomenon, which typically supports immediate performance but is less durable over time (Table 2). Conversely, the lower-intensity usage at Centre 1, where learners described integrating listening into daily routines like commuting, aligns theoretically with ‘spaced practice,’ a strategy known to support retention stability even if immediate gains are more moderate (Tables 6 and 8) [21].

Cognitive load also appears to be a critical factor in the usability of AI-narrated content. Qualitative feedback highlighted that the continuous audio stream, without adequate pauses between questions and answers, may occasionally exceed working memory capacity (Table 8). Participants specifically requested ‘pause-and-recall’ moments to formulate their own responses, a design feature that would support active retrieval practice. The absence of these deliberate pauses in the prototype likely contributed to the ‘passive’ experience reported by some learners, potentially inhibiting the deeper processing required for robust long-term retention (Table 8). Furthermore, the heterogeneity in topic-wise gains suggests that the effectiveness of audio revision is content-sensitive and may depend on the learner’s baseline knowledge ‘headroom’ (Tables 1 and 5).

### Comparison with prior literature

The immediate learning gains observed in this study align with recent systematic reviews indicating that podcasts are effective for knowledge reinforcement in medical education, particularly when used for targeted revision [22]. However, our findings regarding improvement in higher-order application challenge the perception of audio resources as purely passive tools limited to factual recall, suggesting they can support clinical reasoning when scripted effectively [23].

The divergence in retention trajectories may be interpreted through the lens of cognitive psychology. The high-intensity usage at Centre 2 mirrors a ‘massed practice’ phenomenon, whereas the lower-intensity, distributed usage at Centre 1 aligns theoretically with spaced practice, which is known to support retention stability [21]. While previous medical education trials have demonstrated the efficacy of spaced delivery for retention [24], our study highlights how local implementation culture (intensive vs. opportunistic use) can naturally create ‘massed’ or ‘spaced’ conditions with divergent outcomes (Table 2). Furthermore, participant feedback regarding the ‘robotic’ nature of the AI narration resonates with the ‘Voice Principle’ of the Cognitive Theory of Multimedia Learning, which posits that human voices foster deeper social presence and learning than machine-generated narration [10]. Finally, the demonstrated utility of this on-demand revision tool aligns with the National Medical Commission’s Competency-Based Medical Education (CBME) regulations, which explicitly mandate the incorporation of digital resources to foster self-directed learning (SDL) among Indian medical graduates [5, 6].

### Educational and implementation implications

For scalable adoption, these findings suggest that AI-generated podcasts are best positioned as a ‘mobile-first’ revision adjunct rather than a primary learning resource. The clear association between completion rates and learning outcomes (β = 5.15) indicates that implementation strategies should focus on supporting adherence, perhaps through structured listening plans or faculty ‘nudges’ (Table 7; Fig. 1). The stability of retention at Centre 1 suggests that encouraging opportunistic listening (e.g., during commute or chores) is a valid educational strategy that may support long-term retention even if immediate gains are more moderate (Tables 2 and 6).

From a design perspective, the qualitative integration highlights three actionable refinements for future AI-audio tools: (1) Segmentation and Pauses: Deliberate silence should be engineered into the audio tracks-specifically after case scenarios and between questions and answers-to force retrieval practice and reduce cognitive load (Table 8); (2) Multimodal Anchors: Providing a visual companion (e.g., a one-page key-point PDF) may address the ‘friability’ of audio learning and support the revisiting habits learners described (Table 8); and (3) Voice Optimization: Continued refinement of AI prosody is needed to reduce monotony, which was identified as a barrier to sustained attention (Tables 6 and 8).

### Strengths

This study strengthens the evidence base for AI in medical education through its rigorous multicentre mixed-methods design and transparent reporting of participant flow (Fig. 1). Unlike single-site pilots, this study leveraged the geographic diversity of India-spanning centres in the extreme West and East-to test the intervention across distinct institutional cultures, while the standardized National Medical Commission (NMC) curriculum served as a unifying content framework. This dual-context approach allowed for the detection of context-dependent retention patterns that might have been missed in a homogeneous cohort (Tables 1 and 2). The use of linear mixed-effects modelling (LMM) robustly handled missing data and repeated measures, providing a more accurate estimation of trajectories than complete-case analysis alone (Table 2); (Table S1, Additional File 5). Additionally, the triangulation of quantitative predictors with qualitative explanations in the joint display supports coherent meta-inferences about why retention varied, moving beyond simple efficacy testing (Table 8). The consistency of results across parametric and non-parametric sensitivity checks further bolsters confidence in the directional findings (Table 3); (Table S2, Additional File 5).

### Limitations

Inferences must be tempered by several limitations. First, attrition at the delayed follow-up was skewed towards Centre 1, where retention was lower (58.6%) compared to Centre 2 (82.9%), introducing potential selection bias; however, baseline comparisons suggest retained and lost participants were demographically and academically similar (Table 1; Fig. 1). Second, the matching of records using a linkage identifier reduced the analytic cohort for longitudinal comparisons, although reliability and survey analyses utilised the full dataset to maximise representativeness (Fig. 1); Third, ‘dose’ was assessed via self-report, which is subject to recall bias; we mitigated this by using a conservative binary definition (> 50% completion) for the predictor model (Tables 6 and 7). Fourth, the lack of a non-intervention control group means we cannot rule out the influence of concurrent exam preparation (co-intervention), though the centre-specific trajectories and predictor analysis suggest the intervention played a specific role (Tables 2 and 7). Finally, the qualitative insights, while integrated, are drawn from specific institutional contexts and may not fully transfer to settings with different educational cultures (Table 8).

### Future research

Future studies could explore whether objective, individual-level tracking offers greater precision than self-report in defining ‘dose,’ potentially allowing for a more granular analysis of non-linear dose–response relationships (Table 7). Given the ‘pacing’ feedback, experimental comparisons of continuous versus segmented (pause-embedded) audio could empirically test the cognitive load mechanisms hypothesized here (Table 8). Research should also investigate the durability of learning over longer intervals (e.g., 3–6 months) and examine whether structured reinforcement (e.g., spaced ‘recap’ episodes) can arrest the decay observed in high-intensity usage contexts (Table 2); (Table S1, Additional File 5). Furthermore, future iterations could develop podcasts that integrate two or more specialty subjects-such as Medicine, Pediatrics, and Obstetrics & Gynecology-to align with the National Medical Commission’s advocacy for integrated teaching-learning methods and better support students who learn these concurrent subjects simultaneously. Finally, exploring why certain topics (e.g., Hypertension) yielded disproportionately high gains compared to others could inform more nuance in script engineering (Table 5).

## Conclusions

Across two centres, AI-generated, faculty-validated revision podcasts were associated with immediate learning gains in medical students, yielding comparable observations regarding their utility and effectiveness across implementation contexts. While high-intensity usage at one centre drove large immediate gains (dz = 0.55) that were susceptible to decay, more distributed usage at the other was associated with moderate but stable retention. Mixed-methods integration indicates that while learners value the portability of the tool for ‘dead time’ revision, sustained engagement requires design refinements-specifically segmentation, pacing, and multimodal supports-to optimize cognitive load. These findings support the utility of AI podcasts as a flexible, effective revision adjunct, provided implementation considers local usage culture and the need for active reinforcement.

## Supplementary Information

Additional File 1: Reporting Checklist - TREND. File format: .pdf Description: Completed TREND (Transparent Reporting of Evaluations with Nonrandomized Designs) checklist for the quasi-experimental study component.

Additional File 2: Reporting Checklist - GRAMMS. File format: .pdf Description: Completed GRAMMS (Good Reporting of A Mixed Methods Study) checklist for the mixed-methods explanatory sequential design.

Additional File 3: Reporting Checklist - TIDieR. File format: .pdf Description: Completed TIDieR (Template for Intervention Description and Replication) checklist providing a detailed description of the AI podcast intervention.

Additional File 4: Interview Guide and Standard Operating Procedure (SOP). File format: .pdf Description: This file contains the complete semi-structured interview guide used for the Focus Group Discussions, along with the detailed SOP outlining the protocols for moderation, audio recording redundancy, and data handling used to ensure qualitative rigour.

Additional File 5: Supplementary Tables S1–S3.

## Data Availability

The datasets generated and/or analysed during the current study are available in the Figshare repository: https://figshare.com/s/7538eff02c82b04d031e. This repository includes anonymized raw response data, the assessment blueprint mapped to Bloom’s taxonomy, and de-identified qualitative transcripts with the associated framework analysis matrix. The private link is active for peer review and will be made public upon acceptance of the manuscript.
