# Supplementary material for: Ears for examinations: a multicentre quasi-experimental evaluation of AI-generated revision podcasts on learning outcomes and retention among medical students
Source: BMC Med Educ. 2026 Aug 4;26:1532. doi: 10.1186/s12909-026-10080-6 (PMC13613611; doi:10.1186/s12909-026-10080-6)
Supplement: Supplementary file 1 [file 12909_2026_10080_MOESM1_ESM.docx]

**Title**

Ears for Examinations: a multicentre quasi-experimental evaluation of AI-generated revision podcasts on learning outcomes and retention among medical students

# **Author names**:

Urvish Joshi^1^, Amrita Sarkar^2^, Aparajita Shukla^3^, Sheetal Shrimali^1^, Naba Kumar Bezbaruah^2^, Sanjay Kini B^4^*, Sharon Baisil^5^, Zeel Sheth^6^, Pragya Bhargava^1^ and Stuti Shah^1^

## **Additional File 1: TREND checklist**

## **TREND Checklist**

*Transparent Reporting of Evaluations with Nonrandomized Designs* *Used for the quasi-experimental (one-group pre-post) component.*

| **Item No.** | **Descriptor** | **Reported on Page/Section** |
| --- | --- | --- |
| 1 | Title and Abstract  Information on how unit were allocated to interventions (if applicable). Structured abstract recommended. | Title; Abstract |
| 2 | Background  Scientific background and explanation of rationale. Theories used in designing interventions. | Introduction (paragraphs 1–2) |
| 3 | Participants  Eligibility criteria for participants, including criteria at different levels (e.g., cities, clinics, subjects). | Methods - Setting and Participants |
| 4 | Interventions  Details of interventions intended for each study condition and how and when they were actually administered. | Methods - Intervention (The Audio Podcast) |
| 5 | Objectives  Specific objectives and hypotheses. | Introduction (paragraph beginning 'The specific objectives of this study were three-fold…') |
| 6 | Outcomes  Clearly defined primary and secondary outcome measures. Methods used to collect data. | Methods - Outcome Measures |
| 7 | Sample Size  How study size was determined. | Methods - Statistical Analysis - Power Analysis |
| 8 | Assignment Method  Method used to assign units to study conditions (e.g., minimization). | Methods - Setting and Participants |
| 9 | Blinding  Whether participants, providers, and outcome assessors were blinded to assignment. | N/A (Educational intervention; blinding not possible) |
| 10 | Unit of Analysis  Description of the smallest unit that is being analyzed to assess intervention effects. | Methods - Statistical Analysis - Primary Analysis |
| 11 | Statistical Methods  Statistical methods used to compare study groups for primary outcome(s). Methods for additional analyses. | Methods - Statistical Analysis |
| 12 | Participant Flow  Flow of participants through each stage (enrollment, intervention, follow-up, analysis). | Results - Participant flow and dataset completeness; Figure 1 |
| 13 | Recruitment  Dates defining the periods of recruitment and follow-up. | Methods - Setting and Participants |
| 14 | Baseline Data  Baseline demographic and clinical characteristics of participants. | Results - Baseline characteristics (Table 1) |
| 15 | Baseline Equivalence  Data on study group equivalence at baseline. | Results - Baseline characteristics (Table 1) |
| 16 | Numbers Analyzed  Number of participants included in each analysis (denominator). | Results - Participant flow and dataset completeness; Tables 2 and 3 |
| 17 | Outcomes Estimation  Summary of results (point estimates and confidence intervals). | Results - Primary outcome: learning gain and retention (LMM) (Table 2) |
| 18 | Ancillary Analyses  Subgroup analyses and adjusted analysis. | Results - Predictors of learning gain (Table 7) |
| 19 | Adverse Events  Summary of all important adverse events or unintended effects. | N/A (None reported) |
| 20 | Interpretation  Interpretation of results taking into account study hypotheses, bias, etc. | Discussion - Interpretation and mechanisms |
| 21 | Generalizability  Generalizability (external validity) of the trial findings. | Discussion - Strengths; Discussion - Limitations |
| 22 | Overall Evidence  General interpretation of the results in the context of current evidence. | Conclusions |
