# Supplementary material for: Ears for examinations: a multicentre quasi-experimental evaluation of AI-generated revision podcasts on learning outcomes and retention among medical students
Source: BMC Med Educ. 2026 Aug 4;26:1532. doi: 10.1186/s12909-026-10080-6 (PMC13613611; doi:10.1186/s12909-026-10080-6)
Supplement: Supplementary file 2 [file 12909_2026_10080_MOESM2_ESM.docx]

**Title**

Ears for Examinations: a multicentre quasi-experimental evaluation of AI-generated revision podcasts on learning outcomes and retention among medical students

# **Author names**:

## Urvish Joshi^1^, Amrita Sarkar^2^, Aparajita Shukla^3^, Sheetal Shrimali^1^, Naba Kumar Bezbaruah^2^, Sanjay Kini B^4^*, Sharon Baisil^5^, Zeel Sheth^6^, Pragya Bhargava^1^ and Stuti Shah^1^

## **Additional File 2:** GRAMMS checklist

## **GRAMMS Checklist**

*Good Reporting of A Mixed Methods Study* *Used for the explanatory sequential design.*

| **Item** | **Descriptor** | **Reported on Page/Section** |
| --- | --- | --- |
| 1 | Describe the justification for using a mixed methods approach to the research question. | Abstract; Introduction (paragraph beginning 'The specific objectives of this study were three-fold…'); Methods - Study Design |
| 2 | Describe the design in terms of the purpose, priority, and sequence of methods. | Methods - Study Design |
| 3 | Describe each method in terms of sampling, data collection, and analysis. | Methods - Data Collection; Methods - Statistical Analysis; Methods - Qualitative Analysis and Integration |
| 4 | Describe where integration has occurred, how it has occurred, and who has participated in it. | Methods - Qualitative Analysis and Integration; Results - Mixed-methods integration (Joint display) - Table 8 |
| 5 | Describe any limitation of one method associated with the presence of the other method. | Discussion - Limitations |
| 6 | Describe any insights gained from mixing or integrating methods. | Results - Mixed-methods integration (Joint display) - Table 8; Discussion - Interpretation and mechanisms |
