# Supplementary material for: Ears for examinations: a multicentre quasi-experimental evaluation of AI-generated revision podcasts on learning outcomes and retention among medical students
Source: BMC Med Educ. 2026 Aug 4;26:1532. doi: 10.1186/s12909-026-10080-6 (PMC13613611; doi:10.1186/s12909-026-10080-6)
Supplement: Supplementary file 3 [file 12909_2026_10080_MOESM3_ESM.docx]

**Title**

Ears for Examinations: a multicentre quasi-experimental evaluation of AI-generated revision podcasts on learning outcomes and retention among medical students

# **Author names**:

## Urvish Joshi^1^, Amrita Sarkar^2^, Aparajita Shukla^3^, Sheetal Shrimali^1^, Naba Kumar Bezbaruah^2^, Sanjay Kini B^4^*, Sharon Baisil^5^, Zeel Sheth^6^, Pragya Bhargava^1^ and Stuti Shah^1^

## **Additional File 3: TIDieR checklist**

## **TIDieR Checklist**

*Template for Intervention Description and Replication* *Used to describe the AI Podcast intervention.*

| **Item No.** | **Descriptor** | **Details from Manuscript** | **Reported on Page/Section** |
| --- | --- | --- | --- |
| 1 | Brief Name | AI-Generated Revision Podcasts (Ears for Examinations) | Methods - Intervention (The Audio Podcast) |
| 2 | Why | To utilize 'dead time' and reinforce learning via auditory modality. | Introduction; Methods - Intervention (The Audio Podcast) |
| 3 | What (Materials) | Scripts, AI engines (LLMs, Google AI studio - GenAI tools), Adobe Podcast, YouTube channel. | Methods - Intervention (The Audio Podcast) |
| 4 | What (Procedures) | Production, validation, delivery via YouTube/WhatsApp. | Methods - Intervention (The Audio Podcast) |
| 5 | Who Provided | Content curated by Faculty; Audio generated by AI. | Methods - Intervention (The Audio Podcast) |
| 6 | How | Online distribution (WhatsApp); Asynchronous listening. | Methods - Intervention (The Audio Podcast) |
| 7 | Where | Medical College (curation) -> Student Homes/Commute (consumption). | Methods - Intervention (The Audio Podcast) |
| 8 | When and How Much | 6 episodes, 8-10 mins each, over 15-day window. | Methods - Intervention (The Audio Podcast) |
| 9 | Tailoring | Standardized podcasts for all students. | Methods - Intervention (The Audio Podcast) |
| 10 | Modifications | No modifications made during the study period. | N/A |
| 11 | How Well (Planned) | Adherence monitoring via analytics. | Methods - Intervention (The Audio Podcast) - Access and Delivery |
| 12 | How Well (Actual) | Adherence assessed via self-reported frequency and analytics. | Results - Learner perceptions and usage (Table 6) |
