# Supplementary material for: Ears for examinations: a multicentre quasi-experimental evaluation of AI-generated revision podcasts on learning outcomes and retention among medical students
Source: BMC Med Educ. 2026 Aug 4;26:1532. doi: 10.1186/s12909-026-10080-6 (PMC13613611; doi:10.1186/s12909-026-10080-6)
Supplement: Supplementary file 4 [file 12909_2026_10080_MOESM4_ESM.docx]

# Additional File 4: Interview Guide and Standard Operating Procedure

**Study Title: Ears for Examinations: a multicentre quasi-experimental evaluation of AI-generated revision podcasts on learning outcomes and retention among medical students**

**Document Content:**

- **Part A:** Semi-Structured Focus Group Discussion (FGD) Guide
- **Part B:** Standard Operating Procedure (SOP) for Conducting FGDs

## Part A: Semi-Structured Focus Group Discussion (FGD) Guide

**Target Audience:** 3rd Professional MBBS Students who completed the post-test.

**Group Size:** 6-8 participants per session.

**Moderator Role:** Neutral facilitator; probe for honest feedback including negative experiences.

### 1. Introduction (5 Minutes)

- **Opening Statement:** "Welcome, everyone. Thank you for joining us. Today, we want to hear about your honest experience using the AI-generated revision podcasts over the last few weeks. There are no right or wrong answers. We want to know what worked and what didn’t. Your comments are confidential and will be anonymized. This discussion will help us decide if this tool should be used for future batches."

### 2. Theme 1: General Experience & Usage (Icebreaker)

- **Core Question:** "How did it feel to revise Community Medicine using audio instead of just reading books or notes?"
- **Context Probe:** "Think back to the last few weeks. When exactly did you find yourself listening to these episodes? (e.g., while commuting, before sleeping, between classes?)"
- **Barrier Probe:** "Did anyone try to listen but gave up? If so, what stopped you?"

### 3. Theme 2: Content & Cognitive Skills (Higher-Order Learning)

- **Core Question:** "The podcasts included specific case scenarios and problem-solving examples. Did those help you understand the concepts differently than just reading the theory? How?"
- **Cognitive Probe:** "Did you feel the content helped you analyze or apply knowledge, or was it mostly just helping you memorize facts?"
- **Specifics:** "Which specific topic (e.g., Anemia, Malaria, HIV) did you feel was explained best in the podcast format, and why?"

### 4. Theme 3: The ‘AI’ Factor

- **Core Question:** "These scripts were generated by and validated manually by faculties and voiced by AI tools. Did the voice feel natural to you, or was it robotic/distracting?"
- **Attention Probe:** "Did the quality of the audio affect your concentration?"

### 5. Theme 4: Comparison & Confounders

- **Core Question:** "How does this podcast revision compare to watching a YouTube video or reading standard textbooks (e.g., Park, IAPSM, etc.)?"
- **Integration Probe:** "Many of you likely used other methods to study during these few weeks. Did the podcasts replace those methods, or did they just add to them?"

### 6. Closing (5 Minutes)

- **Future Design:** "If we were to make this a permanent part of the curriculum next year, what **one change** would you suggest?"
- **Final Call:** "Any final thoughts or experiences we haven’t covered?"

## Part B: Standard Operating Procedure (SOP) for Conducting FGDs

**Duration:** 30–40 Minutes per session

### 1. Roles & Logistics

- **Seating:** Arranged in a tight circle to facilitate eye contact and discussion. No podiums or desks between the moderator and students.
- **Privacy:** "Research in Progress - Do Not Disturb" signage posted to ensure a quiet environment.
- **Team Composition:**
  - **Moderator (Faculty):** Leads the discussion, manages time, ensures coverage of all themes.
  - **Scribe (Resident/Colleague):** Manages recording devices and documents non-verbal cues (e.g., group consensus, hesitation).

### 2. Audio Recording Protocol (Redundancy Rule)

To prevent data loss, a dual-recording strategy is mandatory:

- **Primary Recorder:** Smartphone placed in the center of the circle on a cloth (to dampen vibration noise).
- **Backup Recorder:** Second device kept by the Scribe/Moderator.
- **Device Settings:** Both devices must be set to **AIRPLANE MODE** to prevent call interruptions.
- **Format:** Standard .m4a or .wav format.

### 3. Moderator’s Checklist

- [ ] Room setup complete (circular arrangement).
- [ ] Airplane Mode ON for all recording devices.
- [ ] Mic Check performed (all voices audible).
- [ ] **Opening Script Read:**
  - "Three Ground Rules: 1. Honesty (we need data, not compliments), 2. Audio Clarity (one speaker at a time), 3. Confidentiality (names removed)."
  - "Do I have your permission to record this session for analysis?" (Verbal consent obtained).
- [ ] Discussion conducted using the Semi-Structured Guide (Part A).
- [ ] **Closing:** Files saved and playback checked immediately.

### 4. Post-Production & Data Handling

- **Debrief (‘Hot Note’):** Immediately post-session, the Moderator and Scribe document:
  - What was the most surprising finding?
  - What was the general mood? (Excited? Bored? Critical?)
- **Transcription Strategy:**
  1. **Initial Draft:** Initial transcription using automated tools.
  2. **Human Verification:** Manual review by a faculty member to correct medical terminology (e.g., ‘Vivax’, ‘Anemia Mukt Bharat’) and ensure accuracy against the original audio.
  3. **Anonymization:** All participant names replaced with codes (e.g., "Student 1", "Student 2") prior to analysis.
