# Supplementary material for: Ears for examinations: a multicentre quasi-experimental evaluation of AI-generated revision podcasts on learning outcomes and retention among medical students
Source: BMC Med Educ. 2026 Aug 4;26:1532. doi: 10.1186/s12909-026-10080-6 (PMC13613611; doi:10.1186/s12909-026-10080-6)
Supplement: Supplementary file 5 [file 12909_2026_10080_MOESM5_ESM.docx]

**Title**

Ears for Examinations: a multicenter quasi-experimental evaluation of AI-generated revision podcasts on learning outcomes and retention among medical students

# **Author names**:

# Urvish Joshi^1^, Amrita Sarkar^2^, Aparajita Shukla^3^, Sheetal Shrimali^1^, Naba Kumar Bezbaruah^2^, Sanjay Kini B^4^*, Sharon Baisil^5^, Zeel Sheth^6^, Pragya Bhargava^1^ and Stuti Shah^1^

# **Additional File 5: Supplementary Tables S1–S3**

## **Table S1. Linear mixed-effects model fixed-effect estimates**

| **Effect** | **Estimate (β)** | **SE** | **95% CI** | **p-value** |
| --- | --- | --- | --- | --- |
| Intercept (Pre-test, Centre 1) | 32.3 | 0.8 | 30.6 to 33.9 | p<0.001 |
| Post (vs Pre) | 3.6 | 0.9 | 1.9 to 5.2 | p<0.001 |
| Delayed (vs Pre) | 2.3 | 1 | 0.4 to 4.3 | 0.018 |
| Centre 2 (vs Centre 1) | 6 | 1.8 | 2.4 to 9.5 | 0.001 |
| Post×Centre 2 | 3.2 | 1.9 | -0.6 to 7.0 | 0.094 |
| Delayed×Centre 2 | 0.4 | 2 | -3.5 to 4.3 | 0.834 |

***Legend:*** *Fixed effects from the primary LMM. Estimates are from REML fit with z-based inference. Planned contrasts with Holm adjustment are reported in Table 2.*

## **Table S2. Sensitivity analysis (paired comparisons)**

| **Analysis** | **Primary method** | **Sensitivity method** | **Agreement (Y/N)** |
| --- | --- | --- | --- |
| Pre vs Post (Centre 1) | Paired t-test | Wilcoxon signed-rank | Y |
| Pre vs Post (Centre 2) | Paired t-test | Wilcoxon signed-rank | Y |
| Post vs Delayed (Centre 1) | Paired t-test | Wilcoxon signed-rank | Directional agreement; significance differed |
| Post vs Delayed (Centre 2) | Paired t-test | Wilcoxon signed-rank | Y |
| Pre vs Delayed (Centre 1) | Paired t-test | Wilcoxon signed-rank | Directional agreement; significance differed |
| Pre vs Delayed (Centre 2) | Paired t-test | Wilcoxon signed-rank | Y |

***Legend:*** *Wilcoxon signed-rank tests were computed for paired total-score comparisons as a robustness check to distributional assumptions. Agreement indicates consistent direction and overall inference pattern relative to paired t-tests.*

## **Table S3. Qualitative themes, subthemes, and exemplar quotes by centre**

| **Theme** | **Subthemes** | **Analytic definition** | **Centre pattern** | **Exemplar evidence (de-identified)** |
| --- | --- | --- | --- | --- |
| Podcasts positioned as a revision tool | Revision focus; Active recall; Complementary (not replacement) | Learners framed the audio as best for quick revision and retrieval practice, not as a substitute for textbooks/first-time learning. | Convergent across both centres. | C1 FGD3, P3: 'It can help for revision.' \| C2, P8: 'podcasts are good for quick revision; YouTube is better for first-time learning, and Park… is still needed for detailed study.' |
| Convenience and multitasking | Travel/chores use; Time efficiency; 'No-pen' revision | Audio enabled revision during otherwise 'dead time' (travel/walking/household tasks), supporting mental rehearsal without writing. | Convergent across both centres; more concrete multitasking examples in Centre 1. | C1 FGD3, P1: 'I was like… traveling.' \| C1 FGD3, P4: 'I listened while walking.' \| C2, P8: 'even in the absence of a pen I could mentally revise the whole chapter.' |
| Engagement and reduced boredom | Less tiring than reading; Makes revision 'interesting' | Audio was experienced as less fatiguing than prolonged reading and sometimes increased willingness to revise. | Convergent across both centres. | C1 FGD2, P2: 'It can make revision more interesting.' \| C2, P3: 'Listening was less boring and tiring than reading Park all the time…' |
| Cognitive engagement through cases | Case scenarios; Practical framing; Knowledge application | Case vignettes were valued for making content feel practical and prompting thinking/application, especially exam-oriented case handling. | Convergent across both centres. | C1 FGD2, P4: 'case scenario was good… It made us think.' \| C2, P1: 'the case scenarios made the topic feel more practical… especially for exams.' |
| Voice and attention | Monotony; Human-like tone; 'Robotic' narration | Sustained attention was shaped by narration style; monotony/robotic tone reduced concentration for some, while others acclimatized to the AI voice. | Convergent across both centres. | C1 FGD2, P2: 'after a point it started getting monotonous and we lost our concentration.' \| C2, P6: 'It mostly felt natural… I didn’t really notice it was AI.' \| C2, P4: 'quite robotic!' |
| Delivery design needs | Pauses; Pacing at key points; Chunking/headings; Summaries | Learners asked for intentional pacing, pauses to process, and clearer structure (segmenting, summarizing, signposting) to reduce cognitive load. | More prominent in Centre 1; partially echoed in Centre 2 via clarity/concentration comments. | C1 FGD1, P1: 'there was no pause in between.' \| C1 FGD1, P1: 'at the important points, the pace should be slower…' \| C2, P2: 'When the sound was clear, my concentration was better.' |
| Trust and credibility | Faculty oversight; Stating sources/standards | Perceived credibility increased when learners believed faculty curated the content and when standard sources were explicitly stated. | Strongly expressed in Centre 1; not a prominent theme in Centre 2 summary. | C1 FGD2, P1: 'it is more trusted because it is made by Professor.' \| C1 FGD1, P2: 'if you can add the source… it will increase the trust in the information.' |
| Platform and usability constraints | YouTube limitations; Note-taking; Background play | Practical constraints of the delivery platform affected usability (screen-on requirement, switching apps for notes, background play limitations). | Centre 1 only (as discussed in FGDs); not covered in Centre 2 framework summary. | C1 FGD2, P1: 'it’s not very convenient… it’s on YouTube… I have to keep my screen on… if I want to put notes…' |

*Legend: Table S3 summarises themes and subthemes derived from three Centre 1 focus group transcripts (FGD-Centre1-01/02/03) and one Centre 2 framework analysis findings summary (FGD-Centre2-01). Themes represent patterned perceptions about the 'Ears for Exams' audio learning resource, including its intended role (revision vs first-time learning), contextual use (multitasking), engagement, cognitive processing, and design/usability considerations. Quotes are brief, de-identified, and labelled by centre and participant identifier as presented in the source documents; ellipses indicate removal of filler words while preserving meaning. Where a theme was not discussed in a centre’s available qualitative materials, this is explicitly stated (e.g., platform constraints in Centre 2 findings summary; trust/credibility emphasis in Centre 2).*
